# State of the Art – Pharynxrekonstruktion

**DOI:** 10.1007/s00106-024-01535-7

**Published:** 2024-12-10

**Authors:** Markus Brunner, Stephan Haerle

**Affiliations:** 1 Karl Landsteiner Privatuniversität für Gesundheitswissenschaften, Dr. Karl-Dorrek-Straße 30, 3500 Krems, Österreich; 2 Klinische Abteilung für Hals‑, Nasen- und Ohrenkrankheiten, Universitätsklinikum Krems, Mitterweg 10, 3500 Krems, Österreich; 3 Zentrum für Kopf-Hals-Chirurgie, Hirslandenklinik St. Anna, 6006 Luzern, Schweiz

**Keywords:** Tumoren, Pharynx, Chirurgische Operationsverfahren, Freie Lappen, Morbidität, Neoplasms, Pharynx, Operative surgical procedures, Free flaps, Morbidity

## Abstract

**Hintergrund:**

Die Rekonstruktion des Pharynx nach ablativer onkologischer Chirurgie ist anspruchsvoll. Die Wiederherstellung der Funktion und die Ästhetik stehen dabei im Mittelpunkt. Es gibt keinen klaren Goldstandard der Rekonstruktion. Die traditionell meist verwendeten freien Lappenrekonstruktionen bedienen sich eines freien Radialis- oder eines freien Oberschenkellappens. Für Defekte mittleren Ausmaßes stellt der freie laterale Armlappen eine hervorragende rekonstruktive Methode dar.

**Ziel der Arbeit:**

Die Vor- und Nachteile der verschiedenen Rekonstruktionsmethoden werden aus der Sicht zweier erfahrener Kopf-Hals-Chirurgen diskutiert und das jeweils übliche Vorgehen bei der Pharynxrekonstruktion beschrieben.

**Schlussfolgerung:**

Die ideale Rekonstruktion hängt von der Größe, vom Volumen des Defekts, von der Gefäßsituation und nicht zuletzt von der Präferenz und Erfahrung der Operateur:innen ab.

## Abstract (en)

**Background:**

Reconstruction of the pharynx after ablative cancer surgery is challenging. Restoration of function and esthetics is at the forefront. There is currently no gold standard for reconstruction. The workhorses for free flap reconstruction are the radial free forearm flap or the anterolateral thigh flap. For medium size defects the free lateral arm flap is an excellent alternative.

**Objective:**

The current article discusses the advantages and disadvantages of the different free flap options from the perspective of two experiences head and neck surgeons and describes the usual steps in pharyngeal reconstruction.

**Conclusion:**

The ideal choice of reconstruction depends on the size and volume of the defect, the availability of donor vessels, and also on the experience and preference of the surgeon.

Im Folgenden wird, aus der subjektiven Sicht der beiden Autoren, deren übliches Vorgehen bei der Pharynxrekonstruktion beschrieben und ihre Lappenpräferenz begründet.

## Ziele der Rekonstruktion

Die Rekonstruktion des Pharynx nach ablativer onkologischer Tumorchirurgie kann sehr herausfordernd sein. Funktion, Design und Ästhetik der Rekonstruktion stehen im Vordergrund – gepaart mit einer möglichst geringen Morbidität im Bereich der Lappenentnahmestelle. Für die Rekonstruktion kommen verschiedene lokale und freie Lappen infrage.

Es gibt keinen klaren Goldstandard

Es gibt keinen klaren Goldstandard. Welcher Lappen für welchen Defekt verwendet wird, hängt von einer Vielzahl an Faktoren ab. Die aktuell international am häufigsten verwendeten freien Lappen für die Pharynxrekonstruktion sind der freie Radialislappen („radial forearm free flap“, RFFF) und der freie laterale Oberschenkellappen („anterolateral thigh flap“, ALT). In den letzten Jahren wurde auch der freie laterale Armlappen („lateral arm flap“, LAF) zunehmend beliebter. Das früher sehr beliebte freie Jejunumtransplantat wird trotz sehr guter funktioneller Ergebnisse [1] nun seltener verwendet, da hierfür das Abdomen eröffnet werden muss und die Morbidität entsprechend steigt. Das dafür notwendige Hinzuziehen von Allgemeinchirurg:innen erhöht zudem die organisatorische Komplexität im chirurgischen Alltag noch mehr.

## „Lateral arm flap“

Der „lateral arm flap“ (LAF) wurde erstmalig 1982 von Song et al. [2] beschrieben. Der fasziokutane Lappen beruht auf der A. collateralis radialis posterior und ihren septokutanen Perforatoren im lateralen intermuskulären Septum des Oberarms. Aufgrund seiner kleinen Gefäßdurchmesser und des eher kurzen Lappenstiels wird der LAF im Vergleich zum RFFF eher selten verwendet, obschon Letzterer mit einer deutlich größeren Morbidität an der Entnahmestelle einhergeht.

Die Anatomie ist in der Mehrheit der Fälle konstant: Die arterielle Versorgung verläuft über die A. collateralis radialis posterior, welche einen Endast der A. brachii profunda darstellt und nahe am Humerusschaft im intermuskulären Septum verläuft. Hiervon werden die Perforatoren zur Haut abgegeben. Der in der Literatur angegeben Arteriendurchschnitt beträgt 1,5 mm [3]. Der venöse Abfluss beruht auf Venae comitantes, welche in 75 % der Fälle doppelt vorhanden sind. Der durchschnittliche Venendurchmesser beträgt 2,5 mm [2]. Der LAF bietet zudem die Möglichkeit, einen sensiblen Nervenast für einen neurovaskulären Hautlappen zu gewinnen.

**Abb. 1:**
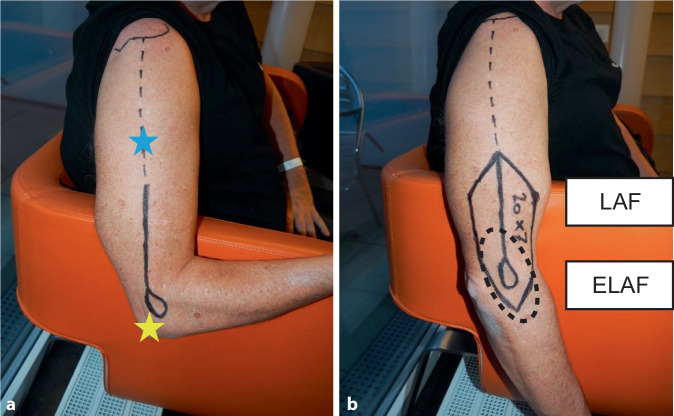
Design eines freien lateralen Armlappens („lateral arm flap“, LAF). **a** Tastbare Anatomie: *Hellblauer Stern* Ansatz des M. deltoideus an der Tuberositas deltoidea, *gelber Stern *Epicondylus humeri lateralis, **b** Einzeichnen der Hautinsel; *LAF* freier lateraler Armlappen, *ELAF* erweiterter freier lateraler Armlappen

**Abb. 2:**
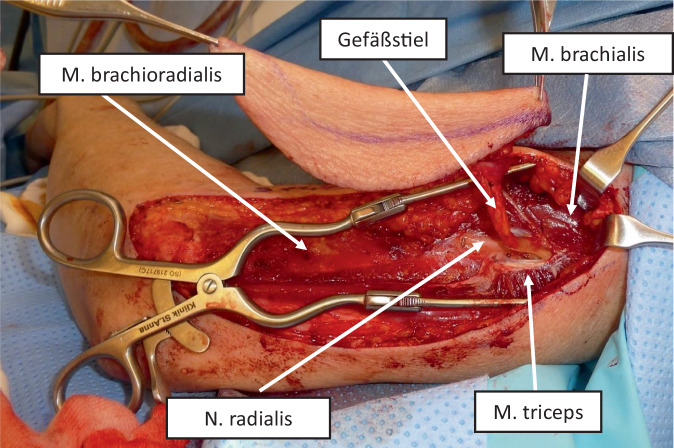
Intraoperativer Situs. Gefäßstiel zwischen M. brachialis anterior und M. triceps posterior. N. radialis in konstanter Lage zwischen M. brachialis und M. brachioradialis. Erläuterung s. Text

**Abb. 3:**
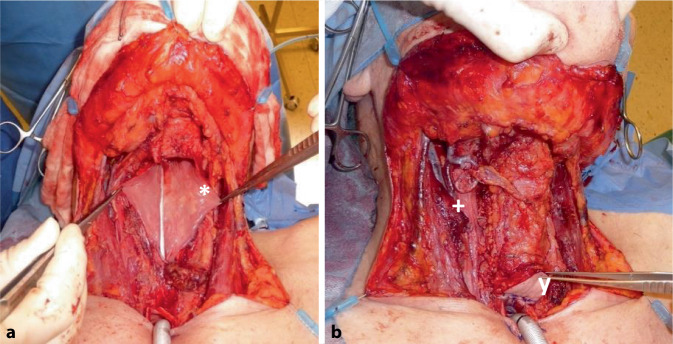
Larynxkarzinom mit laryngopharyngealer Rekonstruktion (inkl. Anbringen einer Monitorinsel) **a** der aufspannte Lappen; *Sternchen* der eingenähte Lappen an der Rachenhinterwand mit der darauf liegenden Nasogastralsonde; **b** der Situs nach Verschluss des Defekts: *+ *vaskuläre Anastomose – *rechts* davon liegt der zu einem Rohr vernähte Lappen; *y *Monitorinsel

Das Heben des LAF wird in den Abb. 1 und 2 gezeigt. Der N. radialis (Abb. 2) muss in allen Fällen dargestellt und geschont werden. Eine Blutsperre ist nicht notwendig; der Hebedefekt kann bis 7 cm Breite primär verschlossen werden. Beispiele einer Pharynxrekonstruktion mittels LAF sind in Abb. 3, 4 und 5 dargestellt.

**Abb. 4:**
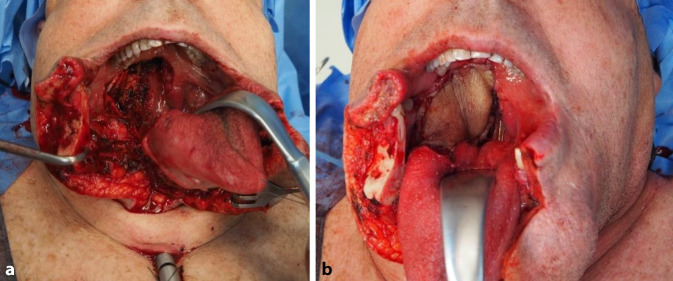
Oropharynxkarzinom mit Rachenhinterwandteilrekonstruktion: **a** Situs nach Tumorresektion mit Kieferspaltung, **b** Situs nach Rekonstruktion der Rachenhinterwand mit freiem Lappen

**Abb. 5:**
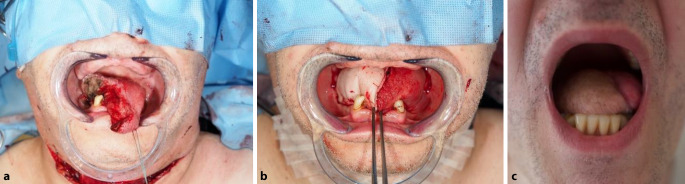
Zungenkarzinom mit Rekonstruktion. **a** Zustand nach Resektion; **b** Zustand nach Einnähen des Lappens; **c** Langzeitverlauf

Die Stärke der vorgestellten Lappenart ist deren Vielseitigkeit. Diese ist anatomisch bedingt: Landmarken zur Lappenhebung sind der Ansatz des M. deltoideus an der Tuberositas deltoidea und der Epicondylus humeri lateralis. Das intermuskuläre Septum mit dem Gefäßstiel liegt 1 cm hinter der Verbindungslinie dieser beiden Landmarken. Neben dem klassischen Oberarmlappen kann der Lappen auch bis maximal 9 cm weiter nach kaudal über den Epicondylus humeri lateralis gezogen werden. Im Englischen spricht man hier vom „extended lateral arm flap“, ELAF. Je weiter man den Lappen nach distal zieht, desto dünner und formbarer wird der Lappen und desto länger der Lappenstiel. Dies erlaubt eine große Flexibilität im Lappendesign.

## Alternative Rekonstruktionstechniken

Das Ziel der Rekonstruktion im Pharynxbereich ist der Verschluss des Schleimhautdefekts, der Schutz der großen Halsgefäße, die Verhinderung von Speichelfisteln und das Erhalten eines für das Schlucken notwendigen Pharynxdurchmessers. Bei kleinen Defekten (bis zu etwa 4 cm) kann dafür ein regionaler Lappen aus der Wange („facial artery myomucosal flap“, FAMM) infrage kommen. Auf eine aufwendige Defektrekonstruktion mittels freier Lappen kann verzichtet werden.

Größere Defekte werden mittels RFFF oder ALT verschlossen

Größere Defekte werden mittels RFFF oder ALT verschlossen. Bei beiden Lappen ist ein simultanes Heben des Lappens während der Ablation möglich. Es gibt kaum hochwertige Studien, welche die Komplikationsraten verschiedener Lappen bei der Pharynxrekonstruktion vergleichen. In Metaanalysen und kleineren Arbeiten gibt es einen Hinweis auf einen besseren Outcome mit RFFF [4, 5].

### „Radial forearm free flap“

**Abb. 6:**
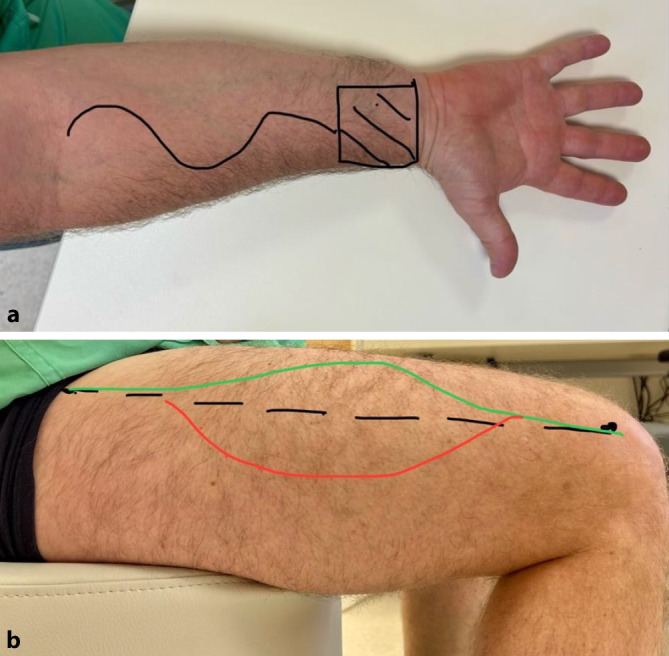
**a** Skizze der Entnahmestelle eines Radialislappens („radial forearm free flap“, RFFF) am Arm, **b** Skizze der Entnahmestelle eines freien lateralen Oberschenkellappens („anterolateral thigh flap“, ALT) am Oberschenkel. *Schwarze Linie *Linie von der Spina iliaca anterior superior zur Patellaoberkante – in der Mitte dieser Linie Lage der meisten Perforatoren; *grüne Linie *initialer Hautschnitt; *rote Linie *Vervollständigung der Definition der Hautinsel erst nach Identifikation der Perforatoren zur Haut

Der „radial forearm free flap“ (RFFF) basiert auf der A. radialis. Die Hautinsel wird unmittelbar proximal zum Handgelenk entnommen (Abb. 6a). Der Vorteil des Radialislappens liegt in seiner geringen Dicke und der dadurch besseren Faltbarkeit, der verlässlichen Durchblutung sowie im langen Gefäßstil mit meist großer Arterie und Vene. Als Nachteil ist v. a. der kosmetisch häufig relativ unschöne Hebedefekt am Arm und die Halbierung der Blutversorgung der Hand zu nennen. Typische Defekte, wo der RFFF zur Defektdeckung herbeigezogen wird, sind Defekte im weichen Gaumen – hier wird der Lappen gefaltet und damit ein vorderes und hinteres Blatt des Gaumens rekonstruiert – und isolierte Defekte der Pharynxseitenwand.

#### Tipps

- Der Allen-Test misst die ausreichende Blutzufuhr der Hand durch die A. ulnaris und ist zwingend notwendig
- Der Hebedefekt kann üblicher Weise mit Vollhaut, die entlang eines geschwungenen Schnitts vom Lappen Richtung Ellenbeuge entnommen wird, gedeckt werden.
- Diese ist kosmetisch besser als Spalthaut.
- Bei der Gaumenrekonstruktion sollte der Lappen mit Nähten durch Bohrlöcher am harten Gaumen befestigt werden, um die häufigen Wunddehiszenzen in diesem Bereich zu minimieren.
- In der Ellenbeuge findet sich fast immer eine große Vene, die den oberflächlichen und tiefen venösen Abfluss des Lappens verbindet, damit kann auf die andernfalls notwendigen 2 venösen Mikroanastomosen verzichtet werden.

### „Anterolateral thigh flap“ (ALT)

Der „anterolateral thigh flap“ (ALT) basiert auf der A. circumflexa femoris und wird am seitlichen Oberschenkel entnommen (Abb. 6b). Nach dem Durchtrennen von Haut und Fascia lata werden aus dem M. rectus femoris kommende Perforatoren gesucht und dann ein verlässlich von diesen Perforatoren versorgtes Haut- und Faszienareal entnommen.

Als Vorteile des Lappens sind

- der große Durchmesser (bis zu etwa 12 × 30 cm) der Hautinsel,
- das mögliche zusätzliche Volumen durch Entnahme von Teilen des M. vastus lateralis und
- die einfache gleichzeitige Entnahme von Fascia lata

zu nennen. Außerdem kann der Hebedefekt praktisch immer primär verschlossen werden und liegt in einem kosmetisch weniger wichtigen Bereich als beim RFFF. Der Gefäßstil ist kürzer als beim RFFF, aber üblicherweise kein limitierender Faktor. Die Morbidität ist bei diesen Eingriffen ein relevanter Faktor – v. a., da es sich meist um ältere Patient:innen mit multiplen Risikofaktoren handelt [6].

Als größter Nachteil muss die manchmal sehr dicke Fettschicht zwischen der Fascia lata und der Haut genannt werden, die den Einsatz des Lappens im Mund und Rachen teilweise unmöglich macht. Aufgrund der Tatsache, dass die hautversorgenden mikroskopischen Perforatoren genau in dieser Schicht liegen, kann diese nicht ausgedünnt werden.

Typische Defekte, bei denen der ALT zum Einsatz kommt, sind voluminöse Defekte im Zungengrund

**Abb. 7:**
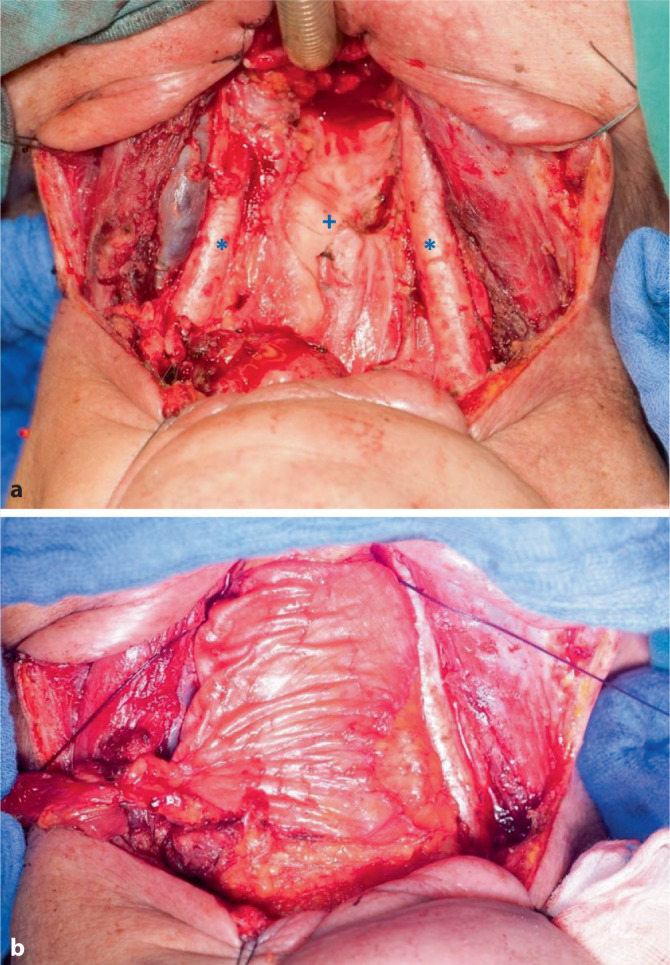
**a** Defekt nach totaler Laryngopharyngektomie, **b** zirkumferente Rekonstruktion mit freiem lateralem Oberschenkellappen („anterolateral thigh flap“, ALT).Erläuterung s. Text. *Sternchen* A. carotis communis, *+ *prävertebrale Faszie

Typische Defekte, bei denen der ALT zum Einsatz kommt, sind voluminöse Defekte im Zungengrund, auch in Kombination mit dem Zungenkörper, oder die Rekonstruktion von Pharynxdefekten nach (Laryngo‑)Pharyngektomie (Abb. 7). Die Haut bildet dabei die Innenauskleidung, die Fascia lata kann als separate Schicht um das Rohr genäht werden.

#### Tipps.

- Der Hautschnitt erfolgt von der Spina iliaca anterior superior nach ventral geschwungen auf das Fibulaköpfchen zu.
- Das genaue Einzeichnen der Hautinsel und der dorsale Schnitt sollte erst nach dem Visualisieren der Perforatoren geplant werden.
- Bei zirkumferenten Pharynxdefekten kann die Fascia lata als zweite Schicht um das Neopharynxrohr genäht werden.

## Fazit für die Praxis

- Das Ziel der Rekonstruktion im Pharynxbereich ist der Verschluss des Schleimhautdefekts, der Schutz der großen Halsgefäße, die Verhinderung von Speichelfisteln und das Erhalten eines für das Schlucken notwendigen Pharynxdurchmessers.
- Ein zufriedenstellendes Ergebnis kann mit verschiedenen Lappen erreicht werden.
- Die Entscheidung, welcher Lappen verwendet wird, hängt nicht nur von Patienten- und Tumorfaktoren ab, sondern auch in großem Ausmaß von der Erfahrung und Präferenz der Operateur:In.
- Die Morbidität ist bei diesen Eingriffen ein relevanter Faktor – v. a., da es sich meist um ältere Patient:innen mit multiplen Risikofaktoren handelt.
